# Integrative analysis reveals novel inflammatory and metabolic pathways in glioblastoma development: A large-scale mediation Mendelian randomization study

**DOI:** 10.1097/MD.0000000000043636

**Published:** 2025-08-01

**Authors:** Xiaolei Yang, Xuejing Lin

**Affiliations:** a Department of Neurosurgery, The First Hospital of China Medical University, Shenyang, China; b Department of Neurology, The First Hospital of China Medical University, Shenyang, China.

**Keywords:** causal inference, glioblastoma, inflammatory factors, Mendelian randomization, metabolic reprogramming, precision medicine

## Abstract

Glioblastoma (GBM) is one of the most lethal central nervous system malignancies, characterized by complex inflammatory responses and metabolic reprogramming. However, the causal relationships between inflammatory factors, metabolites, and GBM risk remain largely unclear. We conducted a 3-stage Mendelian randomization analysis using data from the FinnGen biobank (341 GBM cases and 314,193 controls) and genome-wide association studies of 91 inflammatory factors and 1400 metabolites. For instrument variable selection, we applied a stringent threshold (*P* < 1 × 10^−5^) and performed linkage disequilibrium pruning (*r*^2^ < 0.001). We employed inverse variance weighted method as the primary analysis, supplemented with weighted median, simple mode, and weighted mode methods. Sensitivity analyses included heterogeneity tests, pleiotropy assessments, and leave-one-out analyses. Mediation analysis was performed to explore the metabolic pathways linking inflammatory factors to GBM risk. We identified 4 inflammatory factors causally associated with GBM risk: CCL25 (OR = 1.24, 95% CI = 1.02–1.51), M-CSF1 (OR = 1.70, 95% CI = 1.09–2.68), and IL-33 (OR = 1.61, 95% CI = 1.01–2.56) showed risk-increasing effects, while FGF21 (OR = 0.57, 95% CI = 0.37–0.89) demonstrated protective effects. Among 1400 metabolites, 23 showed significant causal associations with GBM risk across multiple statistical methods. The strongest evidence was found for phospholipid metabolism (1-stearoyl-2-oleoyl-GPE, OR = 1.21, 95% CI = 1.08–1.36) and energy metabolism (cAMP/taurocholate ratio, OR = 0.85, 95% CI = 0.77–0.94). Mediation analysis revealed 21 inflammatory factor-metabolite-GBM pathways, with FGF21 showing the most extensive metabolic regulatory network (12 pathways), followed by M-CSF1 (7 pathways) and CCL25 (2 pathways). Our study establishes a causal framework linking peripheral inflammatory factors and metabolic reprogramming to GBM risk, with FGF21 emerging as a potential protective factor. These findings provide novel therapeutic targets and contribute to the development of precision medicine strategies for GBM.

**Key Points**

- First systematic Mendelian randomization study establishing causal links between inflammatory factors and glioblastoma (GBM) risk.
- Identified 4 key inflammatory factors: CCL25, M-CSF1, IL-33 (risk-increasing) and FGF21 (protective).
- Discovered 23 metabolites significantly associated with GBM risk, particularly in phospholipid and energy metabolism.
- Revealed 21 novel inflammatory factor-metabolite-GBM pathways through mediation analysis.
- FGF21 demonstrated the most extensive metabolic regulatory network with 12 protective pathways.

## 1. Introduction

Glioblastoma (GBM), as one of the most lethal malignancies of the central nervous system, presents significant challenges in understanding its pathogenesis and developing therapeutic strategies.^[1,2]^ Unlike other solid tumors, GBM’s unique anatomical location and the presence of the blood-brain barrier pose special therapeutic challenges. According to recent cancer statistics, although GBM accounts for only about 15% of all brain tumors, it has a mortality rate of up to 95%, with a median survival of just 12 to 15 months.^[3,4]^ In recent years, while significant progress has been made in molecular classification and tumor heterogeneity research through single-cell sequencing and spatial transcriptomics technologies,^[5,6]^ the exact pathogenic mechanisms remain incompletely understood.

The immune microenvironment of the central nervous system exhibits unique characteristics, resulting in inflammatory responses in GBM that differ significantly from peripheral organ tumors. Under normal physiological conditions, the blood-brain barrier strictly controls the entry of peripheral immune cells,^[7]^ but this immune privilege status changes during GBM development and progression.^[8]^ Local inflammatory responses are primarily mediated by microglia and infiltrating macrophages,^[9,10]^ which can secrete various cytokines and chemokines that influence tumor progression. However, our understanding of the causal relationships between specific inflammatory factors and GBM development remains limited.

Metabolic reprogramming in GBM exhibits unique characteristics. Compared to other solid tumors, brain tissue itself has extremely high energy demands and distinctive metabolic patterns. GBM cells not only demonstrate the classic Warburg effect,^[11,12]^ but also show significant glutamine dependency.^[13]^ Notably, due to the selective permeability of the blood-brain barrier, GBM cells’ access to metabolic substrates is strictly limited, prompting them to develop unique metabolic adaptation mechanisms. Recent studies have found that GBM cells can obtain energy substrates through neuron-glial cell metabolic coupling networks,^[14,15]^ providing a new perspective for understanding GBM’s metabolic characteristics.

Mendelian randomization (MR) analysis, as a genomics-based causal inference method, has been widely applied in cancer research in recent years. Compared to traditional observational studies, MR analysis can effectively avoid the influence of confounding factors and reverse causality, providing more reliable evidence for exploring disease mechanisms. Particularly in GBM research, due to the difficulty in obtaining brain tissue samples, MR analysis based on genetic variations provides a unique research strategy for studying the associations between inflammatory factors, metabolites, and disease risk.

This study employs a 3-stage MR analysis strategy to systematically evaluate the causal associations between inflammatory factors, metabolites, and GBM risk. First, based on large-scale GWAS data, we will assess the relationship between 91 circulating inflammatory factors and GBM risk. Second, through analysis of 1400 serum metabolites, we will explore potential metabolic biomarkers. Finally, through mediation MR analysis, we will reveal the molecular mechanisms of the inflammation-metabolism-GBM axis. Unlike previous studies, we pay particular attention to genetic variations in blood-brain barrier-related transporters, which may provide new insights into how peripheral inflammatory factors and metabolites influence GBM development. The findings from this study will help deepen our understanding of GBM pathogenesis and provide theoretical basis for developing new therapeutic strategies.

## 2. Methods

### 2.1. Data sources

This study adopts a multi-source data integration strategy, based on 3 key datasets. The primary data comes from genome-wide association study results from the Finnish FinnGen biobank. This dataset includes genome-wide data from 341 pathologically confirmed glioblastoma patients and 314,193 controls. Considering GBM’s heterogeneity, we specifically selected the dataset identified as C3_GBM_ASTROCYTOMA_EXALLC, which underwent rigorous quality control procedures, excluding other types of brain gliomas to ensure phenotype definition accuracy.^[16,17]^

Regarding metabolomics data, we obtained genome-wide association analysis data for 1400 metabolites covering major metabolic networks through the Genome-Wide Association Studies (GWAS) catalog. These metabolite GWAS studies (ID: GCST90199621-GCST90201020) involve metabolic pathways closely related to brain tissue function, including energy metabolism,^[14]^ neurotransmitter synthesis,^[18]^ and lipid transport.^[19]^ For inflammatory factor data, we included GWAS data for 91 circulating inflammatory factors from the Department of Public Health and Primary Care at Cambridge University (GWAS catalog ID: GCST90274758-GCST90274848).

### 2.2. Selection and validation of instrumental variables

This study strictly follows 3 basic assumptions for instrumental variable selection: genetic variants are significantly associated with exposure factors; genetic variants are not affected by any confounding factors; genetic variants affect outcomes only through exposure factors and not through other pathways. To ensure the validity of instrumental variables, we adopted the following screening strategy:

First, we performed initial screening of GWAS data for 91 inflammatory factors and 1400 metabolites, selecting single nucleotide polymorphism (SNPs) with significance level *P* < 1 × 10^−5^ as candidate instrumental variables. Second, to avoid the influence of linkage disequilibrium, we performed pruning of SNPs using PLINK software,^[20]^ setting the physical distance threshold at 10,000 kb and *R*^2^ threshold at 0.001.

To further ensure the strength of instrumental variables, we calculated the *F*-statistic for each SNP. The *F*-statistic was calculated using the formula: *F* = *R*^2^ × (N-2)/(1 − *R*^2^), where *R*^2^ represents the proportion of exposure variation explained by the SNP, and N is the sample size. *R*^2^ was calculated using the formula: *R*^2^ = 2 × MAF × (1-MAF)×β2, where MAF is the minor allele frequency, and β is the effect value of SNP on exposure. We eliminated SNPs with *F* values <10 to exclude weak instrumental variables.

### 2.3. Statistical analysis

This study employs a 2-sample MR method, comprising 3 main stages:

The first stage evaluates the causal relationships between inflammatory factors and GBM risk. We used the inverse variance weighted (IVW) method as the primary analysis,^[21]^ while employing weighted median method, simple mode method, and weighted mode method for sensitivity analysis. To assess the validity of instrumental variables, we conducted the following tests: using Cochran *Q* test to evaluate effect heterogeneity^[22]^; testing directional pleiotropy through MR-Egger regression intercept^[23,24]^; employing MR-PRESSO method to detect and correct horizontal pleiotropy^[25]^; evaluating the influence of individual SNPs on overall causal effect estimates through leave-one-out analysis.^[26]^

The second stage evaluates the causal relationships between metabolites and GBM risk. Based on the consistency of results across different MR methods, we classified the analysis results into 3 evidence levels: meeting inclusion criteria (statistical significance shown by IVW method only), strong evidence (statistical significance shown by 2–3 methods), and very strong evidence (statistical significance shown by ≥ 4 methods).

The third stage conducts mediation MR analysis to explore the potential mediating role of metabolites in the influence of inflammatory factors on GBM risk. We first evaluated the causal relationships between significant inflammatory factors and metabolites, then assessed the associations between corresponding metabolites and GBM risk, and finally calculated the proportion of mediation effects.^[27]^

For visualization purposes in our forest plots and sensitivity analyses, we applied natural logarithm (ln) transformations to effect estimates to enhance visual clarity, particularly when comparing multiple SNPs with varying effect sizes. This transformation causes values to appear clustered near zero in the visualizations but does not affect the statistical significance or interpretation of the original odds ratios and confidence intervals reported in the text. When interpreting results, we considered both statistical significance (*P* < .05) and the consistency of findings across multiple analytical methods, classifying evidence strength accordingly. For borderline significant results where confidence intervals approach the null, we maintained a conservative interpretation and emphasized findings that demonstrated robustness across sensitivity analyses.

All statistical analyses were performed using R software (version 4.4.1) and related packages (TwoSampleMR, MRInstruments, etc). *P*-values <.05 were considered statistically significant.

## 3. Results

**Figure 1.**
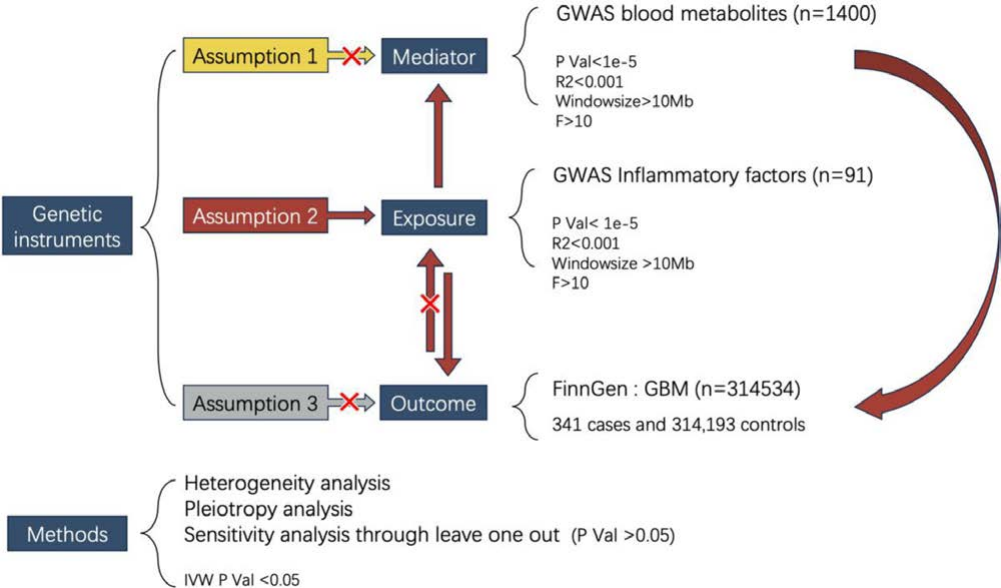
Mendelian randomization analysis process flowchart.

All MR analysis process is shown in Figure 1.

### 3.1. Causal association analysis between inflammatory factors and GBM risk

**Figure 2.**
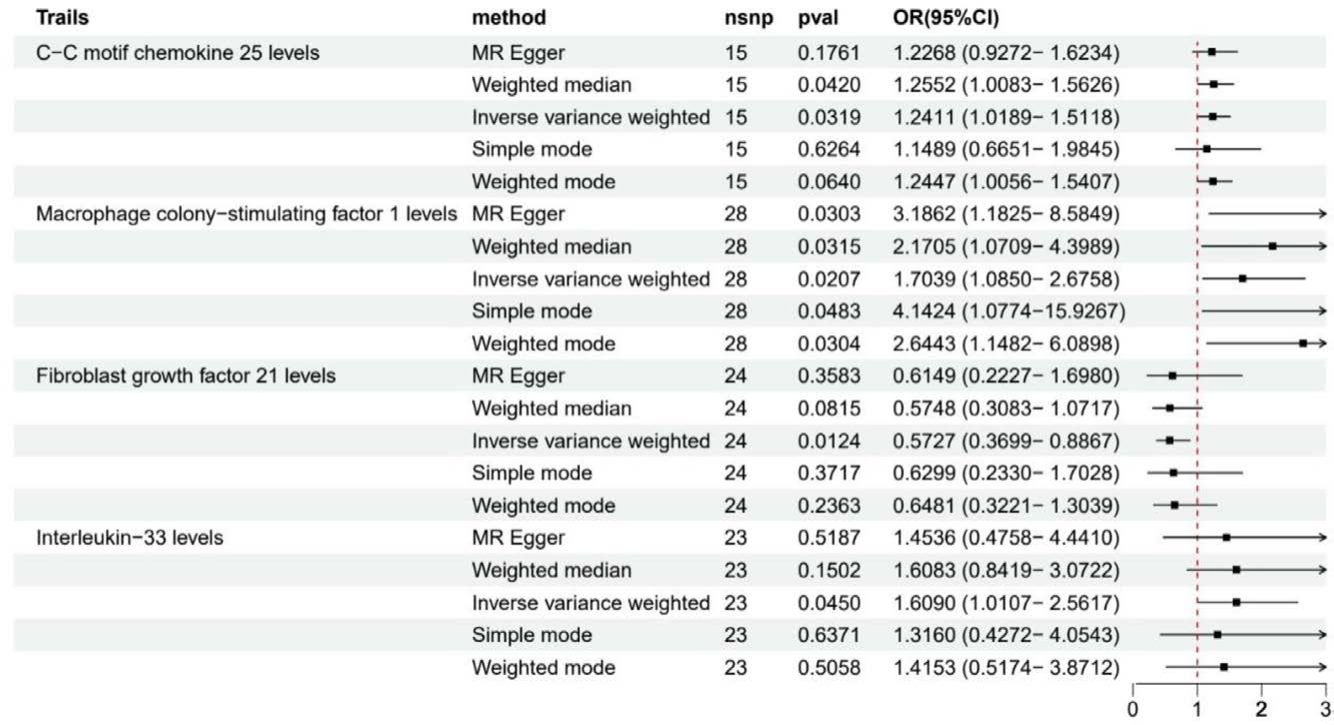
Forest map of MR results of inflammatory factors and GBM. GBM = glioblastoma, MR = Mendelian randomization.

Through 2-sample MR analysis of 91 circulating inflammatory factors, we identified 4 inflammatory factors potentially causally associated with GBM risk (Fig. 2). Results showed that elevated levels of C-C motif chemokine 25 (CCL25, OR = 1.24, 95% CI = 1.02–1.51, *P* = .032), macrophage colony-stimulating factor 1 (M-CSF1, OR = 1.70, 95% CI = 1.09–2.68, *P* = .021) and interleukin-33 (IL-33, OR = 1.61, 95% CI = 1.01–2.56, *P* = .045) were significantly associated with increased GBM risk, while fibroblast growth factor 21 (FGF21, OR = 0.57, 95% CI = 0.37–0.89, *P* = .012) showed a protective effect. Among these, M-CSF1 showed the strongest consistency, with its risk effect supported by all analytical methods, including MR-Egger regression (OR = 3.19, 95% CI = 1.18–8.58, *P* = .030), weighted median method (OR = 2.17, 95% CI = 1.07–4.40, *P* = .032) and weighted mode method (OR = 2.64, 95% CI = 1.15–6.09, *P* = .030).

**Figure 3.**
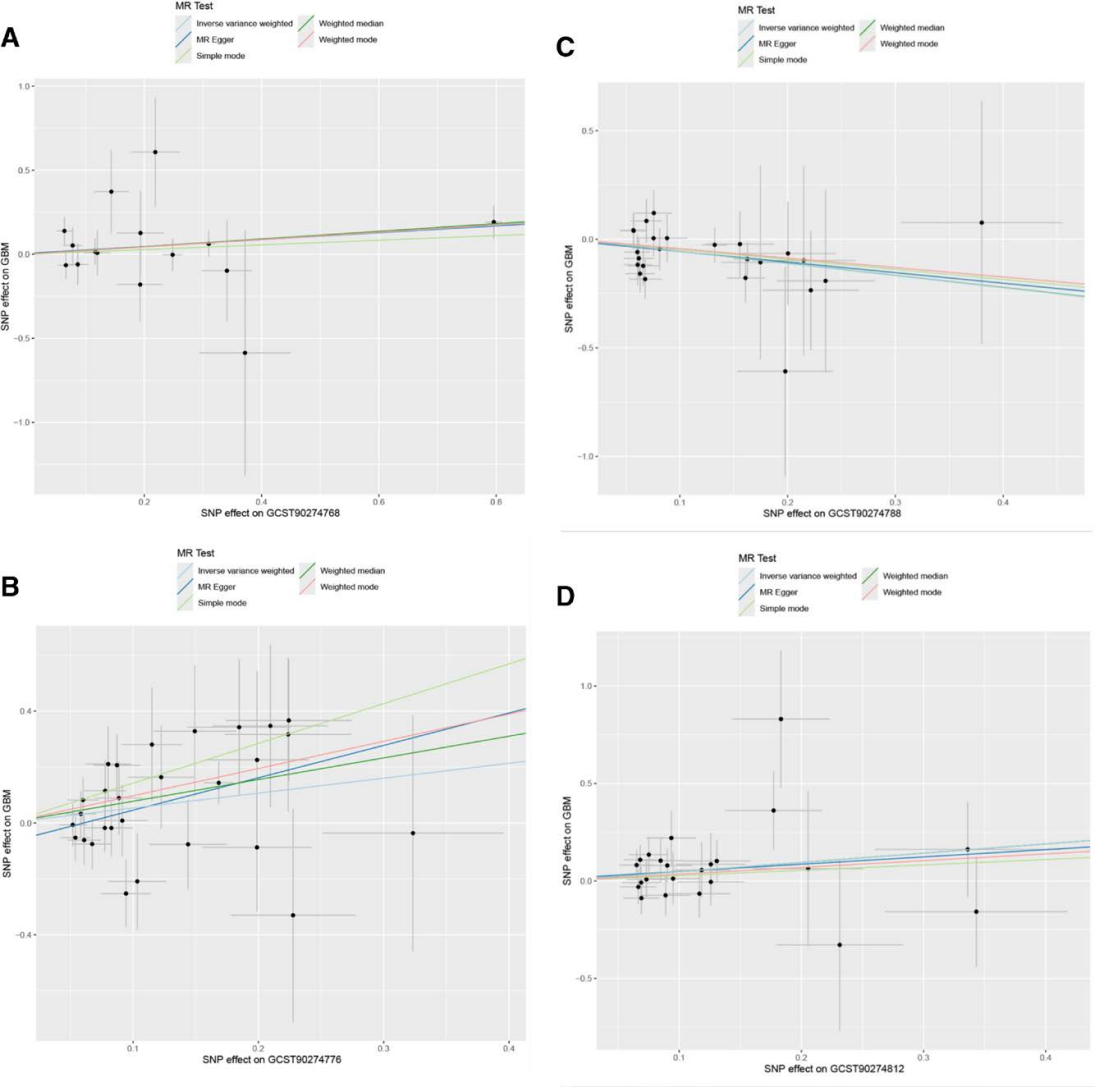
Scatter plots of instrumental variables and MR regression lines for 4 significant inflammatory factors. (A) CCL25 (GCST90274768); (B) M-CSF1 (GCST90274776); (C) FGF21 (GCST90274788); (D) IL-33 (GCST90274812). The x-axis represents the effect of SNPs on inflammatory factor levels, and the y-axis represents the effect of SNPs on GBM risk. Different colored lines represent different MR analysis methods. GBM = glioblastoma, MR = Mendelian randomization, SNPs = single nucleotide polymorphisms.

To verify the robustness of these findings, we conducted systematic sensitivity analyses. Scatter plot analysis (Fig. 3) showed that CCL25, M-CSF1, and IL-33 exhibited positive slopes, while FGF21 showed a negative slope, with regression lines consistent across various MR methods.

**Figure 4.**
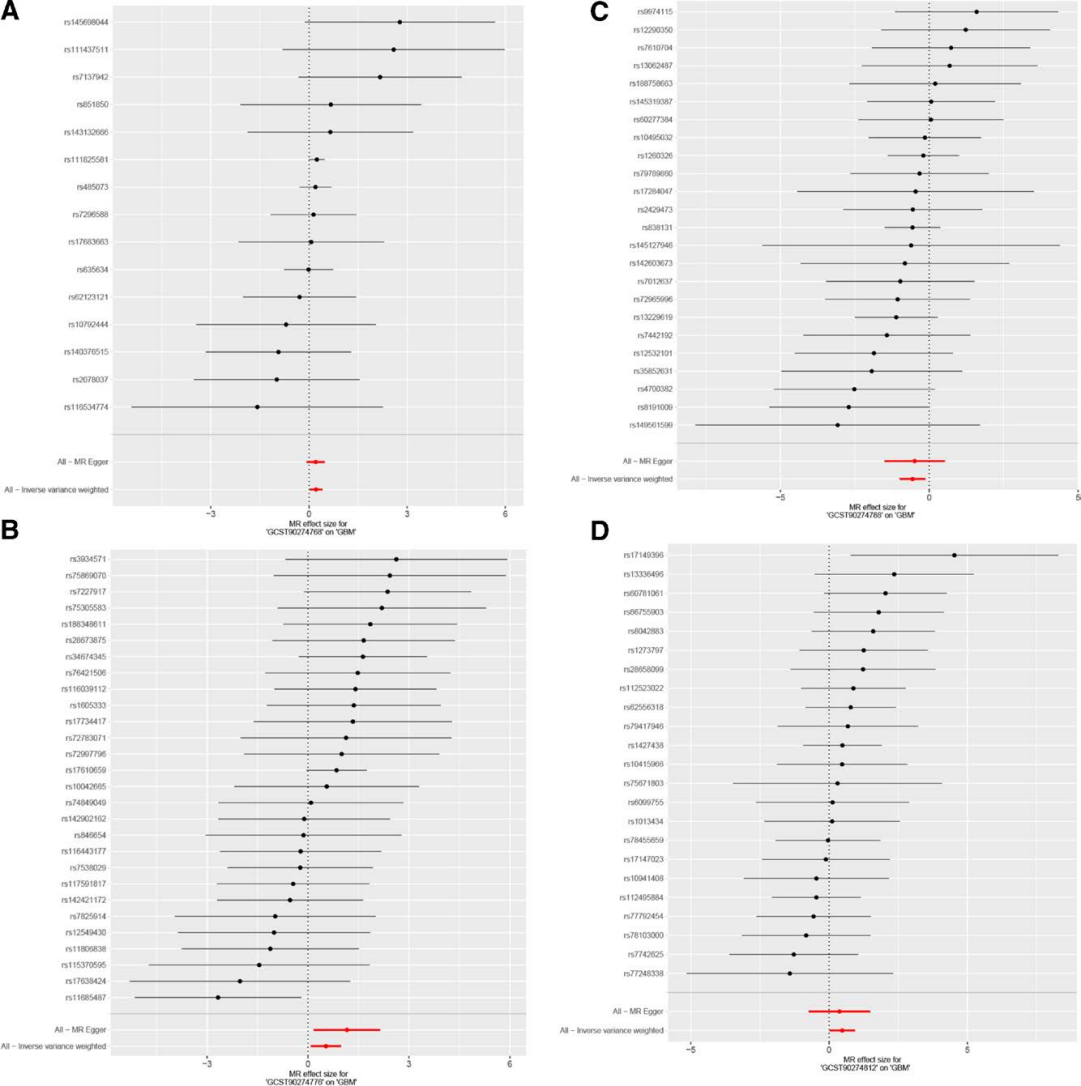
Forest plot analysis of SNP effects for 4 inflammatory factors. (A) CCL25 (GCST90274768); (B) M-CSF1 (GCST90274776); (C) FGF21 (GCST90274788); (D) IL-33 (GCST90274812). SNP = single nucleotide polymorphism.

In single-SNP analysis (Fig. 4), we identified several key genetic instrumental variables: rs111825581 for CCL25 (OR = 1.27, 95% CI = 1.00–1.61, *P* = .049) showed significant risk effect, while rs8191009 (OR = 0.07, 95% CI = 0.01–0.97, *P* = .047) and rs4700382 (OR = 0.08, 95% CI = 0.01–1.22, *P* = .069) for FGF21 showed protective effects. Heterogeneity tests showed no significant effect heterogeneity for any factors (CCL25: *Q* = 11.34, *P* = .659; M-CSF1: *Q* = 28.00, *P* = .411; FGF21: *Q* = 15.38, *P* = .881; IL-33: *Q* = 19.06, *P* = .642). In pleiotropy testing, MR-Egger regression intercepts for all factors were close to zero and not statistically significant, further supporting the validity of instrumental variable selection.

**Figure 5.**
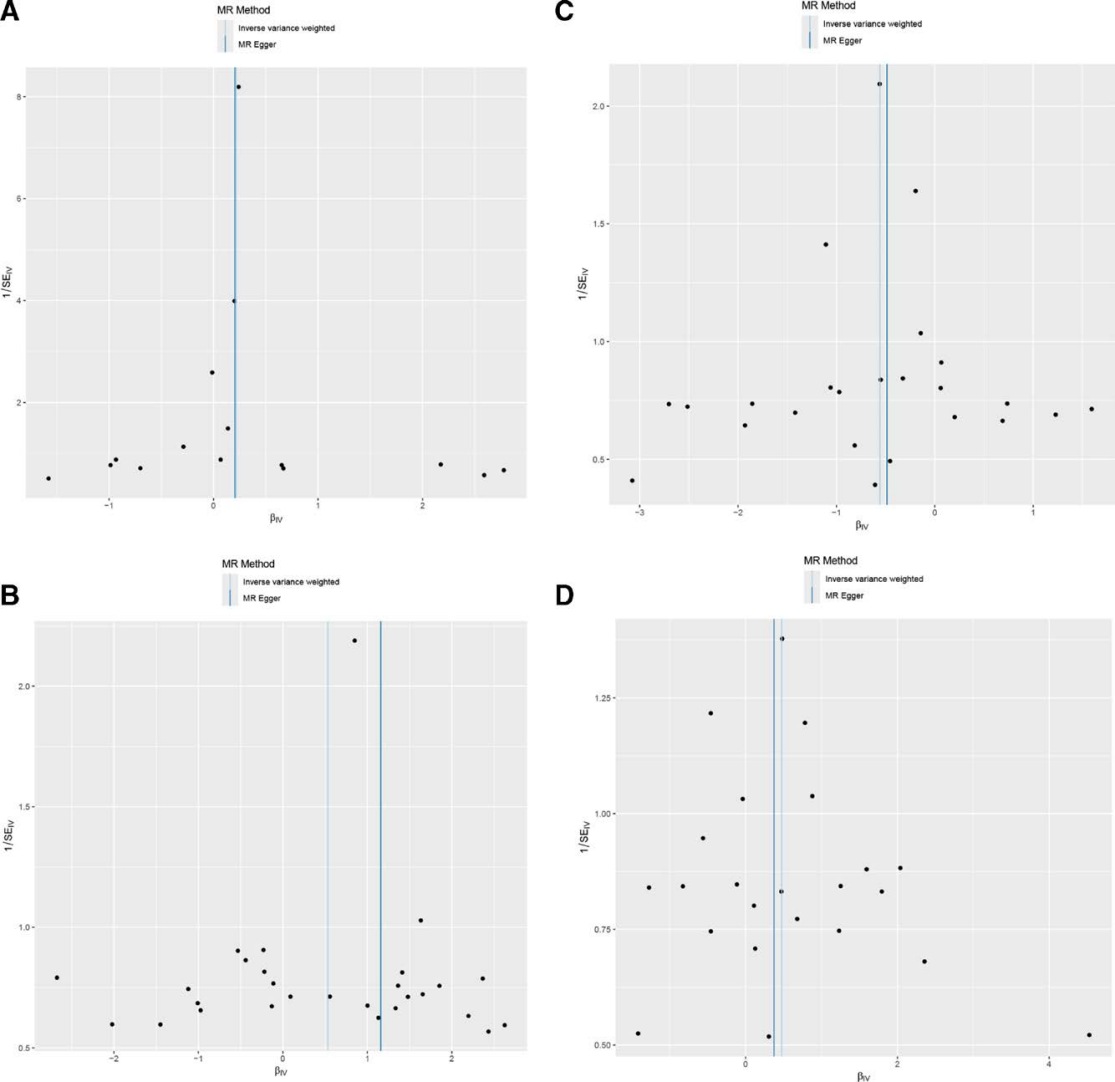
Funnel plots of MR analysis for 4 inflammatory factors. (A) CCL25 (GCST90274768); (B) M-CSF1 (GCST90274776); (C) FGF21 (GCST90274788); (D) IL-33 (GCST90274812). MR = Mendelian randomization.

**Figure 6.**
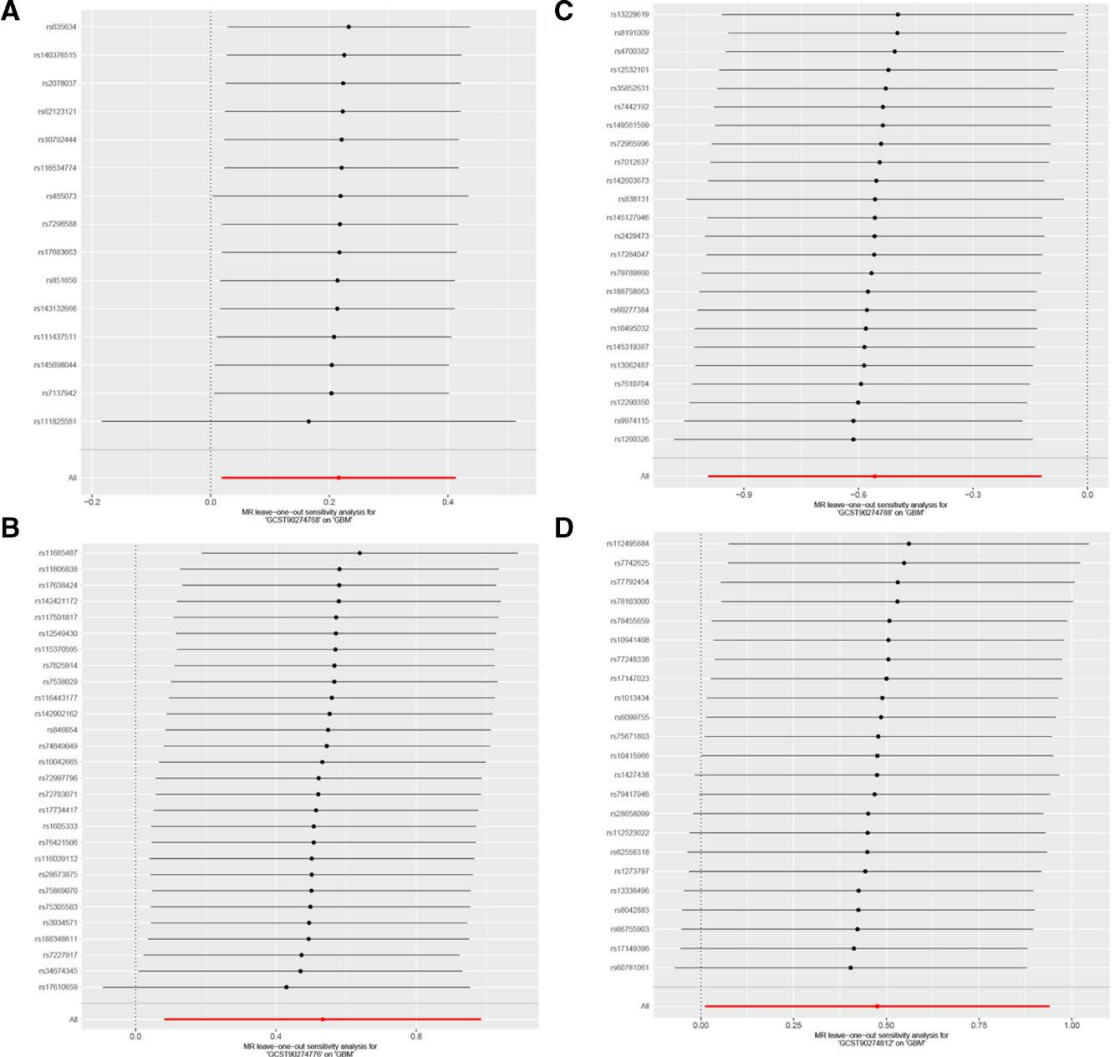
Leave-one-out sensitivity analysis for 4 inflammatory factors. (A) CCL25 (GCST90274768); (B) M-CSF1 (GCST90274776); (C) FGF21 (GCST90274788); (D) IL-33 (GCST90274812). Points and line segments represent effect estimates and 95% confidence intervals after removing corresponding SNPs. SNPs = single nucleotide polymorphisms.

Funnel plot analysis (Fig. 5) showed that except for IL-33, SNP effect estimates for the other 3 factors demonstrated good symmetry, suggesting no significant publication bias. Leave-one-out sensitivity analysis (Fig. 6) indicated that the causal effect estimates for each factor remained stable after removing any single SNP, with M-CSF1 and FGF21 results showing the strongest robustness.

All significant inflammatory factors’ instrumental variables demonstrated sufficient strength (mean *F*-statistics > 10), and no obvious weak instrument bias was found. These comprehensive sensitivity analysis results support our main findings that M-CSF1, IL-33, and CCL25 may serve as risk factors for GBM, while FGF21 may have a protective effect.

**Figure 7.**
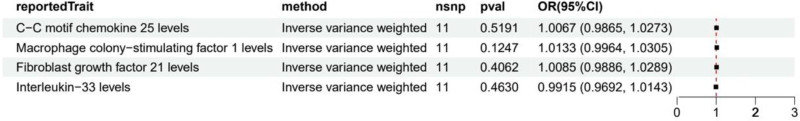
Reverse MR analysis of inflammatory factors and GBM risk. GBM = glioblastoma, MR = Mendelian randomization.

Additionally, to verify the direction of causality, we conducted reverse causation analysis (Fig. 7). Results showed no significant causal associations between GBM and levels of CCL25 (OR = 1.01, 95% CI = 0.99–1.03, *P* = .519), M-CSF1 (OR = 1.01, 95% CI = 1.00–1.03, *P* = .125), FGF21 (OR = 1.01, 95% CI = 0.99–1.03, *P* = .406), and IL-33 (OR = 0.99, 95% CI = 0.97–1.01, *P* = .463). These results rule out the possibility of GBM reversely affecting these inflammatory factor levels, further supporting the directionality of our observed causal relationships.

### 3.2. Assessment of the causal relationship between metabolites and GBM

**Figure 8.**
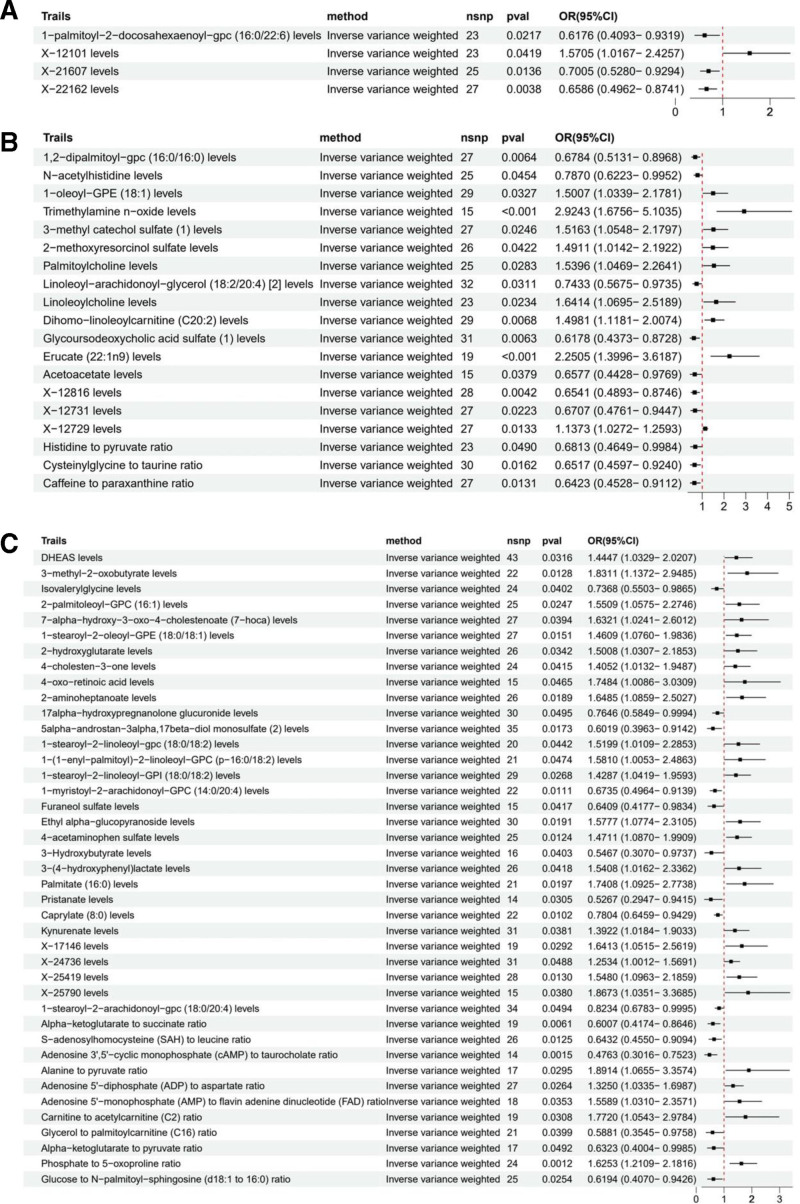
Mendelian randomization analysis of metabolites and GBM risk. (A) Metabolites showing significance (*P* < .05) across all statistical analyses. (B) Metabolites showing significance (*P* < .05) in multiple statistical methods including IVW. (C) Metabolites showing significance (*P* < .05) in IVW method only. GBM = glioblastoma, IVW = inverse variance weighted.

Through MR analysis of 1400 metabolites, we classified significant results into 3 major evidence levels based on consistency across different MR methods (Fig. 8). Among these, 4 metabolites showed significant associations across all statistical analysis methods, providing the strongest evidence support (Fig. 8A):

1. Elevated levels of 1-palmitoyl-2-docosahexaenoyl-gpc (16:0/22:6) showed protective effects (IVW: OR = 0.62, 95% CI = 0.41–0.93, *P* = .022)
2. Elevated X-12,101 levels were significantly associated with increased GBM risk (IVW: OR = 1.57, 95% CI = 1.02–2.43, *P* = .042)
3. Elevated X-21,607 levels demonstrated protective effects (IVW: OR = 0.70, 95% CI = 0.53–0.93, *P* = .014)
4. Elevated X-22,162 levels showed protective effects (IVW: OR = 0.66, 95% CI = 0.50–0.87, *P* = .004)

Another 19 metabolites reached significance in multiple statistical methods including IVW, providing moderate-strength evidence (Fig. 8B). Among these, the most significant included Erucate (22:1n9), where elevated levels were significantly associated with increased GBM risk (IVW: OR = 2.25, 95% CI = 1.40–3.62, *P* = .001), supported by MR-Egger method (OR = 3.34, 95% CI = 1.21–9.21, *P* = .032), weighted median method (OR = 2.01, 95% CI = 1.03–3.93, *P* = .040) and weighted mode method (OR = 2.63, 95% CI = 0.91–7.56, *P* = .090). Elevated trimethylamine n-oxide levels were significantly associated with increased GBM risk (IVW: OR = 2.92, 95% CI = 1.68–5.10, *P* < .001), showing consistent risk effects across all analysis methods. Elevated dihomo-linoleoylcarnitine (C20:2) levels were significantly associated with increased GBM risk (IVW: OR = 1.50, 95% CI = 1.11–2.01, *P* = .007), and received support in other analysis methods. Elevated Linoleoylcholine levels were significantly associated with increased GBM risk (IVW: OR = 1.64, 95% CI = 1.07–2.52, *P* = .023), with other analysis methods also showing consistent risk effects.

### 3.3. Assessment of the causal relationship between circulating inflammatory factors and metabolites

**Figure 9.**
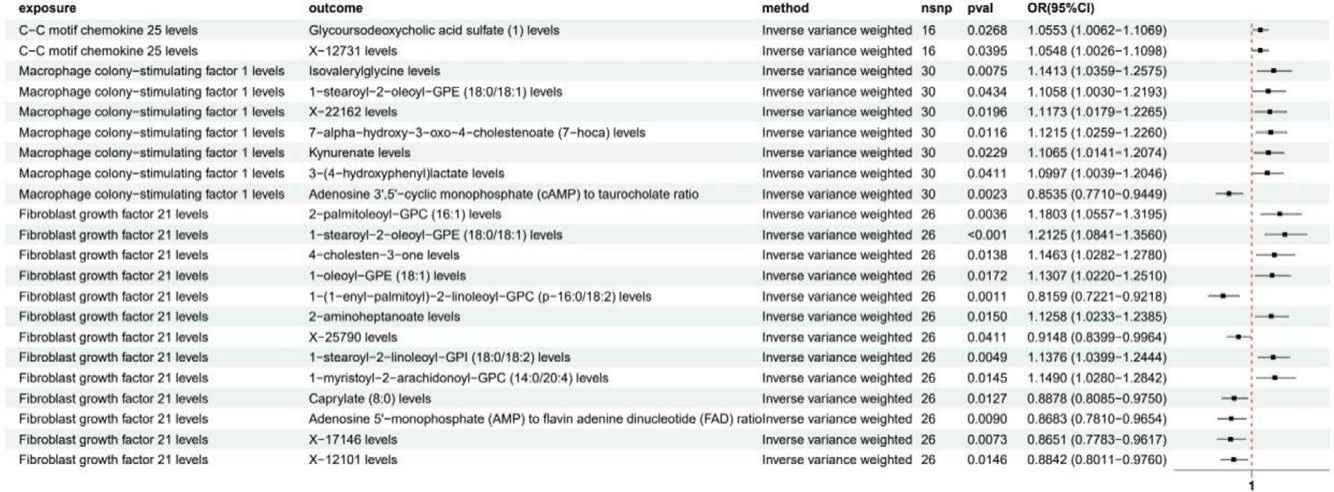
Forest plot of MR analysis for the causal effect of inflammatory factors on metabolites. MR = Mendelian randomization.

Through systematic mediation analysis, we identified 21 potential inflammatory factor-metabolite-GBM pathways (Fig. 9). Among these, CCL25 mediated 2 pathways, M-CSF1 mediated 7 pathways, and FGF21 mediated 12 pathways, suggesting different inflammatory factors may influence GBM development through distinct metabolic pathways.

In CCL25-mediated pathways, we found it could participate in disease progression by affecting levels of glycoursodeoxycholic acid sulfate (OR = 1.06, 95% CI = 1.01–1.11, *P* = .027) and X-12,731 (OR = 1.05, 95% CI = 1.00–1.11, *P* = .040). This suggests CCL25 may primarily act through bile acid metabolism pathways.

For M-CSF1, we found it exhibited more extensive metabolic regulatory effects. The most significant relationships were through Isovalerylglycine (OR = 1.14, 95% CI = 1.04–1.26, *P* = .008) and 7-alpha-hydroxy-3-oxo-4-cholestenoate (OR = 1.12, 95% CI = 1.03–1.23, *P* = .012). Notably, M-CSF1 showed significant negative correlation with cyclic adenosine monophosphate (cAMP)/taurocholate ratio (OR = 0.85, 95% CI = 0.77–0.94, *P* = .002), suggesting it may act through influencing cell signal transduction and bile acid metabolism.

FGF21 demonstrated the most complex metabolic regulatory network, involving 12 different metabolites. The most significant positive correlation was with 1-stearoyl-2-oleoyl-GPE (OR = 1.21, 95% CI = 1.08–1.36, *P* < .001), while the most significant negative correlation was with 1-(1-enyl-palmitoyl)-2-linoleoyl-GPC (OR = 0.82, 95% CI = 0.72–0.92, *P* = .001). These findings highlighted that FGF21 primarily acts through regulating phospholipid metabolism and lipid metabolism pathways. Notably, many of these metabolites are involved in neuronal membrane components, suggesting FGF21 may participate in GBM pathogenesis by influencing cell membrane lipid composition.

## 4. Discussion

### 4.1. Mechanisms of inflammatory factors in GBM pathogenesis

Through MR analysis, this study systematically evaluated the causal associations between inflammatory factors and GBM risk for the first time, identifying 4 key inflammatory factors potentially involved in GBM pathogenesis through different mechanisms, including CCL25, M-CSF1, IL-33, and FGF21. Among these, M-CSF1 showed the strongest promoting effect (OR = 1.70, 95% CI = 1.09–2.68), consistent with its central role in tumor-associated macrophage polarization.^[28,29]^ In the GBM microenvironment, M-CSF1 may promote tumor progression through several pathways: first, it promotes monocyte differentiation into M2-type macrophages, which possess tumor-promoting and immunosuppressive properties^[30,31]^; second, M-CSF1 may directly promote glioma cell proliferation and invasion through activation of PI3K/AKT and MAPK signaling pathways^[32,33]^; additionally, our mediation analysis showed M-CSF1 is associated with multiple metabolites, suggesting it may act by reshaping the tumor metabolic microenvironment.

While CCL25, as an important member of the chemokine family,^[34,35]^ its role in GBM pathogenesis has not been fully understood. Our study found that elevated CCL25 levels were significantly associated with increased GBM risk (OR = 1.24, 95% CI = 1.02–1.51), echoing recent research on chemokines’ roles in the tumor microenvironment.^[36]^ CCL25 may act by recruiting CCR9-positive immune cells to the tumor microenvironment, including regulatory T cells and myeloid-derived suppressor cells, thereby promoting the formation of an immunosuppressive microenvironment. Particularly when blood-brain barrier integrity is compromised, the recruitment of peripheral immune cells may accelerate disease progression.

The promoting effect of IL-33 (OR = 1.61, 95% CI = 1.01–2.56) may be related to its role in glial cell activation.^[37]^ As an alarming family member, IL-33 plays important roles in central nervous system injury responses.^[38,39]^ In the GBM environment, IL-33 secreted by activated astrocytes may stimulate tumor cell proliferation through the ST2 receptor, while promoting angiogenesis and matrix remodeling.^[40]^ Notably, IL-33 may also reshape the local immune environment by recruiting type 2 innate lymphoid cells (ILC2s).^[41,42]^

However, unlike the other 3 promoting factors, FGF21 showed significant protective effects (OR = 0.57, 95% CI = 0.37–0.89). This finding is particularly notable as FGF21 is primarily considered a metabolic regulator.^[43]^ Our analysis showed FGF21 is significantly associated with 12 metabolites, suggesting it may exert protective effects through regulating tumor energy metabolism. Additionally, FGF21 may inhibit GBM progression through suppressing inflammatory responses, improving mitochondrial function, and promoting autophagy.

The blood-brain barrier plays a crucial role in these inflammatory factor-mediated effects. Under normal physiological conditions, the blood-brain barrier strictly controls the entry of peripheral immune molecules and cells. However, our research suggests these inflammatory factors may participate in disease progression by affecting blood-brain barrier permeability. In particular, CCL25 and IL-33 may promote disease progression by compromising blood-brain barrier integrity, facilitating peripheral immune cell infiltration and inflammatory factor penetration.

### 4.2. Role of metabolic reprogramming in GBM development

Among all 1400 metabolites, we identified 64 metabolites with significant causal associations with GBM risk, of which 23 demonstrated stronger causal associations across multiple statistical methods, revealing the specific metabolic characteristics of GBM.

First, lipid metabolism reprogramming shows particular importance in GBM development. We found multiple phospholipid metabolites, such as 1-stearoyl-2-oleoyl-GPE (18:0/18:1) (OR = 1.21, 95% CI = 1.08–1.36) and 1-(1-enyl-palmitoyl)-2-linoleoyl-GPC (OR = 0.82, 95% CI = 0.72–0.92) significantly associated with GBM risk. This association has important biological significance: first, these phospholipids are important components of neuronal membranes, and their changes may affect membrane fluidity and signal transduction; second, certain phospholipids can act as bioactive molecules directly participating in cell signal transduction; furthermore, changes in phospholipid metabolism may reflect tumor cells’ demand for membrane structure remodeling, consistent with GBM’s highly invasive characteristics.^[44]^

Second, in terms of energy metabolism, we observed significant metabolic restructuring. Particularly, the decrease in cAMP/taurocholate ratio (OR = 0.85, 95% CI = 0.77–0.94) suggests changes in adenylate cyclase signaling pathways. This change may affect multiple key cellular processes: first, decreased cAMP levels may affect PKA-mediated signaling pathways,^[45]^ thereby regulating cell proliferation and survival; second, changes in energy metabolism may reflect tumor cells’ adaptation to the Warburg effect^[46]^; additionally, we also found significant changes in AMP/FAD ratio, suggesting mitochondrial function alterations, consistent with the metabolic reprogramming characteristics of GBM cells.

Interestingly, the association between bile acid metabolism and GBM is an unexpected finding of this study. Elevated glycoursodeoxycholic acid sulfate levels were significantly associated with increased GBM risk, suggesting potential unrecognized roles of bile acid signaling pathways in GBM development. Bile acids and their derivatives may influence GBM progression through the following mechanisms: first, they may act as signaling molecules regulating cellular homeostasis; second, changes in bile acid metabolism may reflect liver-brain axis dysregulation^[47]^; additionally, certain bile acids may affect cell proliferation and apoptosis through nuclear receptor signaling pathways.

Moreover, amino acid metabolism reprogramming shows unique characteristics. We found that amino acid metabolites such as Isovalerylglycine and 2-aminoheptanoate were significantly associated with GBM risk. This association may reflect multiple important biological processes: first, changes in amino acid metabolism may be related to imbalances in protein synthesis and degradation; second, certain amino acids may affect the tumor microenvironment as neurotransmitter precursors; additionally, changes in branched-chain amino acid metabolism may be related to tumor cell energy supply. Particularly, the observed changes in kynurenate levels (mediated through M-CSF1) suggest alterations in the tryptophan metabolism pathway, which may be related to the formation of an immunosuppressive microenvironment.^[48]^

Additionally, these metabolic changes do not exist independently but form a highly integrated network. For example, we found that FGF21 may exert protective effects by regulating multiple metabolites, suggesting coordinated regulation of metabolic networks. Meanwhile, these metabolic changes are closely related to the actions of inflammatory factors, forming complex feedback regulatory networks. Understanding such complex metabolic networks is crucial for developing new therapeutic strategies, particularly in metabolic-targeted therapy.

### 4.3. Integrated analysis of the inflammation-metabolism-GBM axis

The CCL25-mediated metabolic pathways exhibit unique characteristics. We found that CCL25 mainly acts through 2 metabolic pathways: the glycoursodeoxycholic acid sulfate pathway (OR = 1.06, 95% CI = 1.01–1.11) and the X-12,731 pathway (OR = 1.05, 95% CI = 1.00–1.11). This relatively simple metabolic network may reflect CCL25’s specific function as a chemokine. Notably, both pathways are related to bile acid metabolism, suggesting that CCL25 might influence blood–brain barrier permeability by regulating bile acid metabolism, thereby promoting immune cell infiltration. This finding provides a new perspective for understanding how chemokines influence the tumor microenvironment through metabolic reprogramming.

M-CSF1 demonstrates a more complex metabolic regulatory network involving 7 significant metabolic pathways. Among these, the association with isovalerylglycine is most significant (OR = 1.14, 95% CI = 1.04–1.26), suggesting the importance of amino acid metabolism in M-CSF1-mediated effects. Additionally, M-CSF1 shows significant correlations with metabolites such as 7-alpha-hydroxy-3-oxo-4-cholestenoate and kynurenate, reflecting the complex metabolic requirements in regulating tumor-associated macrophage function. Particularly, the negative correlation with cAMP/taurocholate ratio (OR = 0.85, 95% CI = 0.77–0.94) suggests that M-CSF1 might influence macrophage polarization states through regulating energy metabolism and signal transduction.

FGF21’s metabolic network is the most extensive and complex, involving 12 different metabolites. This complexity likely stems from FGF21’s central role as a metabolic regulator. In terms of positive regulation, FGF21 shows significant correlation with phospholipid metabolism, particularly the elevation of 1-stearoyl-2-oleoyl-GPE (OR = 1.21, 95% CI = 1.08–1.36), which may reflect important changes in cell membrane components. In terms of negative regulation, FGF21’s significant negative correlation with 1-(1-enyl-palmitoyl)-2-linoleoyl-GPC (OR = 0.82, 95% CI = 0.72–0.92) suggests selective regulation of phospholipid metabolism. This broad yet selective metabolic regulation might explain FGF21’s protective effects.

Interactions between multiple pathways exhibit complex network characteristics. First, we observed that different inflammatory factors might act through common metabolites, such as M-CSF1 and FGF21 both being closely related to lipid metabolism pathways. Second, certain metabolites might serve as hub molecules between different pathways, such as bile acid metabolites being regulated by both CCL25 and M-CSF1. Moreover, these pathways might form feedback regulatory loops, where FGF21-mediated metabolic changes might in turn affect the expression and function of inflammatory factors.

More importantly, this complex network might explain certain clinical phenomena in GBM treatment. For example, the limited effectiveness of single-target therapy might be related to this complex metabolic-inflammatory network, as blocking a single pathway might activate compensatory mechanisms. Meanwhile, the existence of such networks also suggests the necessity of combination therapy, particularly the potential for better therapeutic outcomes when targeting both inflammatory and metabolic pathways simultaneously.^[49]^

### 4.4. Research value, limitations and future prospects

Overall, this study innovatively integrated multi-level omics data to systematically evaluate the causal associations between inflammatory factors and GBM, and revealed potential metabolic regulatory mechanisms. This research strategy has several significant advantages and innovations, while also having some limitations, providing important implications for future research.

In terms of research advantages, first, this is the first use of MR to systematically evaluate causal associations between 91 inflammatory factors and GBM risk, effectively avoiding confounding factors present in traditional observational studies. Second, through integrated analysis of GWAS data for 1400 metabolites, it provides the most comprehensive metabolic profile of GBM to date. Third, it innovatively employs mediation analysis to reveal 21 potential inflammation-metabolism-GBM signaling pathways, providing a systematic perspective for understanding disease mechanisms. Furthermore, the methodological innovations, particularly in handling blood-brain barrier-related factors, provide important reference for future similar studies.

When comparing our findings with prior observational studies, we observe both consistencies and novel insights. For instance, Lin et al^[50]^ recently conducted a 2-sample MR study investigating the causal relationship between cytokines and GBM risk. While their study identified certain inflammatory factors associated with GBM broadly, our research specifically focuses on GBM and extends the analysis to include a more comprehensive panel of 91 inflammatory factors. Furthermore, our integration of metabolomic data provides mechanistic insights not available in previous studies. Where Lin et al identified associations with several interleukins, our findings regarding IL-33 add to this growing body of evidence suggesting cytokine involvement in GBM. However, our identification of CCL25, M-CSF1, and particularly the protective role of FGF21 represents novel contributions to the field. The divergence in some findings may reflect the heterogeneity of GBM subtypes, with our focus specifically on GBM providing greater phenotypic precision. These complementary approaches collectively strengthen the evidence for inflammatory pathway involvement in GBM development while highlighting the need for subtype-specific analyses.

However, this study has several limitations that need to be acknowledged. First, the selection of instrumental variables may be limited by existing GWAS sample sizes and population composition, particularly for some rare inflammatory factors where instrumental variable strength might be insufficient. Second, the accuracy of GBM phenotype definition may be affected by disease heterogeneity, potentially impacting causal inference precision. Third, research results are primarily based on European population data, requiring further validation in other ethnic groups. Additionally, this study uses cross-sectional data, unable to reflect the dynamic disease development process, limiting our understanding of temporal changes.

From a clinical translation perspective, the findings of this study have important implications. Regarding biomarkers, our discovered 4 key inflammatory factors (particularly M-CSF1 and FGF21) and related metabolites could serve as disease prediction and monitoring markers. For therapeutic targets, the results suggest multiple potential intervention targets, especially FGF21-mediated protective pathways that might provide directions for developing new therapeutic strategies. In terms of prevention strategies, deep understanding of inflammatory factors and metabolic pathways helps formulate more targeted preventive measures. Furthermore, these findings provide new insights for achieving precision medicine in GBM, particularly in developing individualized treatment plans.

Based on this study’s findings and limitations, future research needs to focus on several aspects: first, functional validation studies are needed, particularly experimental verification of key inflammatory factors and metabolites’ mechanisms of action. Second, multi-omics integration analysis should be expanded to include epigenomic, proteomic, and other data for a more comprehensive understanding. Third, rigorous clinical translation studies are needed to validate these findings’ application value in clinical practice. Finally, it is necessary to conduct multi-ethnic population studies to confirm these findings’ universality and explore race-specific differences. Therefore, despite some limitations, this study provides important scientific discoveries through innovative research strategies. These findings not only deepen understanding of GBM pathogenesis but also provide important guidance for future research and clinical practice. Future research should focus on validating these findings and advancing their clinical translation, with the ultimate goal of improving GBM patient prognosis.

## 5. Conclusion

In this comprehensive study, we employed a 3-stage MR approach to systematically investigate the causal relationships between inflammatory factors, metabolites, and GBM risk. Our analysis identified 4 key inflammatory factors (CCL25, M-CSF1, IL-33, and FGF21) causally associated with GBM risk, with M-CSF1 showing the strongest promoting effect and FGF21 exhibiting significant protective properties. Through metabolomic analysis, we discovered 23 metabolites significantly associated with GBM risk, particularly highlighting the importance of lipid metabolism and energy metabolic reprogramming in GBM development. Furthermore, our mediation analysis revealed 21 potential inflammatory factor-metabolite-GBM pathways, providing novel insights into the complex interplay between inflammation and metabolism in GBM pathogenesis.

## Author contributions

**Conceptualization:** Xiaolei Yang, Xuejing Lin.

**Data curation:** Xiaolei Yang.

**Formal analysis:** Xiaolei Yang.

**Investigation:** Xiaolei Yang.

**Supervision:** Xuejing Lin.

**Validation:** Xuejing Lin.

**Writing – original draft:** Xiaolei Yang.

**Writing – review & editing:** Xuejing Lin.
